# New Method for the Union of Wounds

**Published:** 1847-10

**Authors:** 


6. *New Method for the Union of Wounds.*—M. Baudens, surgeon in chief at the "Val-de-Grace," addresses a letter to the Academy, in which he presents a method for uniting wounds which he has recently discovered, and which he daily practises with complete success at the hospital of "Val-de-Grace." This simple and efficient method is not, according to M. Baudens, liable to the same objections as adhesive plasters and sutures. The following is the author's description of his method:

"If we have to unite the flaps, resulting from a tibio-tarsal amputation, we fix in the bandage carried circularly above the amputation two strong pins, the one in front and the other behind, taking care to leave their heads and points free. The middle of a long cotton thread is now passed like a noose under the free ends of the pins. The threads are then brought down so as to cross each other upon the flaps approximated by the fingers of an aid, and carried up to the pin of the opposite side, to be again brought down so as to operate as a uniting bandage as often as may be necessary, sometimes parellel with the axis of the limb and sometimes crossing each other so as to form a figure of 8. The threads ligating the arteries are also attached to the pins so as not to be torn away when the dressing is removed. The cotton threads exercise a gentle pressure, they are not easily impregnated by liquids, and may maintain their position a long time. The air and the spaces between them permit the humors of the wound to flow readily, and the traction they exert upon the circular bandage placed above the amputation tends to bring down the flesh and to prevent its forming a cone."

This mode of union is applicable to all wounds in general, but it is necessary to know how to place suitably the bandages for the pins. M. B. succeeded remarkably in thus effecting a lineal and prompt union of the wound resulting from the removal of a large wen from the head.—Translated from *Ibid*, for *Ibid*.

